# Diagnosis and Treatment of a Type III Dens Invagination Using Cone-Beam Computed Tomography

**DOI:** 10.22037/iej.2016.17

**Published:** 2016

**Authors:** Mohsen Bahmani, Alireza Adl, Samane Javanmardi, Sina Naghizadeh

**Affiliations:** a * Postgraduate Student, Department of Endodontics, Dental School, Shiraz University of Medical Sciences, Shiraz, Iran; *; b *Department of Endodontics, Biomaterial Research Center, Dental School, Shiraz University of Medical Sciences, Shiraz, Iran*

**Keywords:** Cone-Beam Computed Tomography, Dens Invagination, Maxillary Lateral Incisor

## Abstract

A 20-year-old man presented with the history of pain and swelling in the anterior maxillary segment. The periapical radiography was indicative of a dental anomaly in right maxillary lateral incisor. Due to the insufficient information from conventional radiography, cone-beam computed tomography (CBCT) was ordered. CBCT showed apical root resorption, large apical lucency and two separate canals with distinct apical foramen (Oehlers type *III* dens invagination). The CBCT image was used as a guide for dentine removal with an ultrasonic tip. Conventional root canal therapy was done using lateral compaction technique. One-and two-year follow-up radiographies revealed periapical repair and absence of symptoms.

## Introduction

Dens invaginatus or “dens in dente” is a well-recognized dental anomaly that may result in pulp necrosis or periradicular pathosis. Maxillary lateral incisor is the most common tooth involved in this anomaly followed by maxillary central incisors [1, 2]. Although the etiology of this anomaly is unknown and controversial, it is generally accepted that this developmental defect is due to the uncontrolled growth of enamel epithelium into the tooth germ [3, 4].

The classification of dens invaginatus by Oehlers is the most common system used in the clinical studies. This categorization is based on the depth of penetration and communication with periodontal ligament and periapical tissues [4]. *Type I* is the minor form and the invagination is within the crown. In *type II*, the invagination extends beyond the cemento-enamel junction and it may or may not communicate with the dental pulp. In *type III*, the invagination penetrates through the root and forms a second apical or lateral foramen without any communication with the pulp [5, 6].

Dens invaginatus is usually detected serendipitously in the radiography, but the conventional radiographic imaging is incapable of demonstrating the exact anatomical feature of the invagination due to its two dimensional representation of a three-dimensional structure [7]. Cone-beam computed tomography (CBCT) is a useful system for accurate dental and maxillofacial evaluations. CBCT produces three-dimensional images of the anatomy of the teeth and their surrounding tissues with reduced radiation exposure to patient compared to conventional tomography [8]. 

There are several treatment options for this anomaly according to clinical symptoms [9]. The treatment plan can vary from a prophylactic fissure sealant in an asymptomatic tooth or conventional root canal therapy in pulpal involvement to complicated therapies such as apical surgery or intentional replantation. Should all other treatments fail, extraction would be the last choice [10].

This report documents the treatment of a challenging case of dens invaginatus of a permanent maxillary lateral incisor with a two year follow-up, indicative of successful outcome.

**Figure 1:**
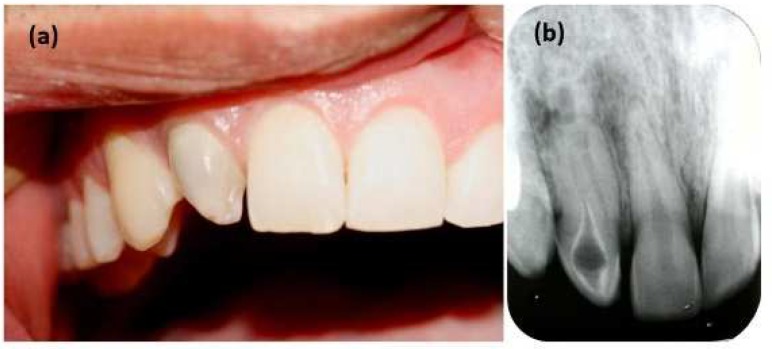
Preoperative; *A)* clinical image and; *B)* radiography of the maxillary right central incisor

## Case Report

A 20-year-old male was referred to the Department of Endodontics, Shiraz Dental School for endodontic treatment of his right maxillary lateral incisor. Medical and familial histories were noncontributory. He reported a previous history of pain and swelling in anterior maxillary area. On clinical examination, the crown of tooth #7 was dome-shaped with slight discoloration (Figure 1A). The periodontal probing depths were less than 3 mm. Thermal and electrical pulp tests did not elicit any responses. Periapical radiography of the right maxillary lateral incisor revealed an invaginated tooth with two separated canals (Oehlers’ *type III* dens invagination) associated with a periapical lesion (Figure 1B). Approximately one-third of the invagination seemed to be lined by enamel. As the morphology of the invagination was not clear on the periapical radiography, CBCT was ordered for optimal diagnosis and treatment protocol. The CBCT images were obtained by a small volume scan (Kodak 9000 3D, Kodak Dental Systems, Carestream Health, Rochester, NY, USA) at 90 kVp, 6 mA, for10 sec.

**Figure 2:**
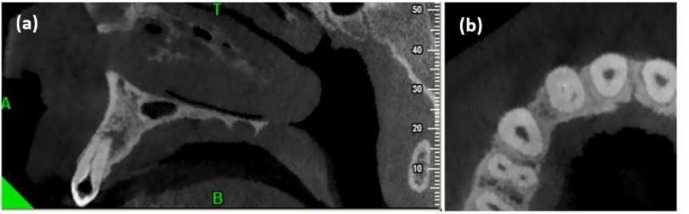
*A)* Labial view showing cross bite of maxillary central incisors and left lateral incisor and; *B)* Palatal view of the same tooth

The CBCT scans revealed that the periapical radiolucency was larger than what was seen on radiography. Two separate canals with distinct apical foramen were observed. The invaginated canal was almost at the center of the root but the other canal was located mesiolingually. The coronal part of the second canal was obliterated. Root resorption associated with the invaginated canal was also observed (Figure 2).

After administration of local anesthesia [buccal infiltration of 2% lidocaine containing 1:80000 epinephrine (Darupakhsh, Tehran, Iran)] and isolation of the tooth with rubber dam, the endodontic access cavity was prepared. The invaginated canal was easily found and negotiated however the second canal was not located due to obliteration of the coronal third. Using the CBCT image as a guide, ultrasonic tips were utilized for dentin removal. Care was taken not to remove excessive dentin. Finally the second canal was located. The working length was determined using Root ZX apex locator (J. Morita USA, Inc., Irvine, CA, USA) and radiography. 

Both canals were instrumented using ProTaper rotary files (Dentsply Maillefer, Ballaigues, Switzerland).

**Figure 3:**
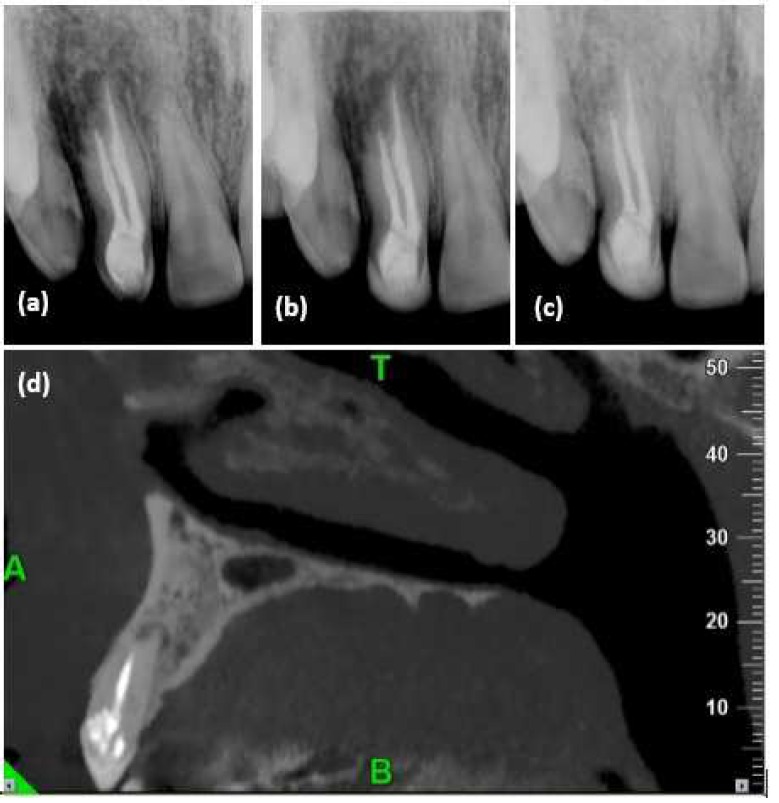
Postoperative radiographies; *A)* immediately after treatment; *B*) one-year and *C and D*) two-year follow ups

Irrigation was done with 2.5% sodium hypochlorite. After finishing the preparation, the root canals were filled with calcium hydroxide paste and the teeth was temporarily restored. After two weeks root canals were copiously irrigated with 2.5% sodium hypochlorite to remove calcium hydroxide dressing. The canals were subsequently dried and filled using lateral compaction technique with gutta-percha and AH-26 sealer (Dentsply, Tulsa Dental, Tulsa, OK, USA). The crown was restored with light-cured composite resin Solitaire 2 (Heraeus Kulzer, Wehrheim, Germany). The patient was recalled 1 and 2 years later. The tooth was asymptomatic and apical repair was observed as the size of the periapical lesion had decreased significantly in both CBCT and periapical radiographies (Figure 3).

## Discussion

This study represents a complicated case of Oehlers *type III* dens invaginatus in a maxillary right lateral incisor with two separated root canals associated with a large periapical lucency and root resorption, as confirmed by CBCT images. Periapical radiographies have limitation in revealing the type, extension and complex morphology of dens invaginatus compared to CBCT. CBCT as an advanced imaging technique helps in the diagnosis, treatment plan and follow-up with this developmental anomaly [8].

In our case no communication was clinically observed between the invagination and oral cavity, but it must have been present since a periapical lesion had developed. Moreover, the invaginated canal did not have any communication with the main canal. As the regular canal was also nonvital, therefore probability of retrograde infection causing pulp necrosis should be considered. Different treatment modalities including nonsurgical root canal treatment, apexification, surgical procedures or even extraction are available for treatment of *type* *III* invagination [4]. Planning a treatment protocol depends on the internal anatomy, the condition of the pulp and the stage of root development [9]. 

Some authors have reported treating the invaginated canal yet retaining pulp vitality in the second canal [2, 11, 12]. In our case, this was not possible because both canals were nonvital. Therefore, the decision was to treat both canals. Root canal treatment of invaginated teeth is usually challenging because of complex variations of root canal morphology or due to difficult access to the regular and invaginated canals [13]. This was also true in the current case. After access cavity preparation, the invaginated canal was easily found while the other canal, with coronal obliteration, was not detected. Therefore CBCT images was used as a guide and ultrasonic tips were utilized for dentin removal. CBCT is able to produce undistorted three-dimensional images of the teeth and has great benefit in the localization and identification of root canals. In the current case, CBCT helped us to locate the obliterated canal without excessive dentin removal. In the present case the role of CBCT cannot be over looked; it is unlikely that endodontic treatment could have been performed as accurately and safely as it was without the aid of the CBCT images. Due to the irregularities and unusual anatomy of the root canal system and the presence of root resorption, in this case, the canals were irrigated copiously with sodium hypochlorite and dressed with calcium hydroxide. The absence of signs and symptoms and healing of periapical lesion after two years confirmed the successful treatment of this case.

## Conclusion

CBCT provides more details of the internal anatomy of dens invagination and is a useful adjunct in the endodontic treatment of this developmental dental anomaly.
